# Aldose reductase inhibitor increases doxorubicin-sensitivity of colon cancer cells and decreases cardiotoxicity

**DOI:** 10.1038/s41598-017-03284-w

**Published:** 2017-06-09

**Authors:** Himangshu Sonowal, Pabitra B. Pal, Jian-Jun Wen, Sanjay Awasthi, Kota V. Ramana, Satish K. Srivastava

**Affiliations:** 1 Department of Biochemistry and Molecular Biology, University of Texas Medical Branch, Galveston, TX-77555 USA; 2 Department of Microbiology and Immunology, University of Texas Medical Branch, Galveston, TX-77555 USA; 3 Department of Internal Medicine, Texas Tech University Health Sciences Center, Lubbock, TX-79430 USA

## Abstract

Anthracycline drugs such as doxorubicin (DOX) and daunorubicin remain some of the most active wide-spectrum and cost-effective drugs in cancer therapy. However, colorectal cancer (CRC) cells are inherently resistant to anthracyclines which at higher doses cause cardiotoxicity. Our recent studies indicate that aldose reductase (AR) inhibitors such as fidarestat inhibit CRC growth *in vitro* and *in vivo*. Here, we show that treatment of CRC cells with fidarestat increases the efficacy of DOX-induced death in HT-29 and SW480 cells and in nude mice xenografts. AR inhibition also results in higher intracellular accumulation of DOX and decreases the expression of drug transporter proteins MDR1, MRP1, and ABCG2. Further, fidarestat also inhibits DOX–induced increase in troponin-I and various inflammatory markers in the serum and heart and restores cardiac function in mice. These results suggest that fidarestat could be used as adjuvant therapy to enhance DOX sensitivity of CRC cells and to reduce DOX-associated cardiotoxicity.

## Introduction

Despite recent advances in diagnostic, surgical, and therapeutic techniques, colorectal cancer (CRC) remains one of the leading causes of death among cancer patients, mostly from recurrent and metastatic disease^1, 2^. Although recent advances in understanding the molecular basis of cancer have provided insight into the network of signaling pathways that regulate cancer cell growth and metastasis, effective long-term therapy for most cancers remains elusive^3, 4^. Unlike normal cells, cancer cells undergo modifications that help them bypass the normal control of cell growth and proliferation^5, 6^. Changes in signaling pathways in response to oxidative stress caused by carcinogens such as heavy metals, environmental pollutants, cigarette smoke, toxic gasses, and ionizing radiation are pleiotropic and have been well-described to participate in carcinogenesis. Reactive oxygen species (ROS) levels below a certain limit elicit cancer cell growth and proliferation while an excess of ROS induces cancer cell apoptosis^7,8]^. Further, oxidative stress causes a significant increase in protein kinase C **(**PKC), phosphoinositide 3-kinase (PI3K), phospholipases A2 (PLA2) and other enzymes that activate nuclear factor kappa- B (NF-κB) and activating protein-1 (AP-1)^7, 9^. The activated NF-κB and AP-1 transcribe various inflammatory markers, growth factors, chemokines, and cytokines, which cause toxicity as well as contribute to the abnormal cell growth observed in cancer^10^.

Chemotherapy is one of the most common therapeutic options for colon cancer, specifically at the advanced stages and after surgical removal of the tumor^11^. Although this process is to a great extent effective, aggressive treatment can be limited by the severe side effects associated with high doses of chemotherapeutic drugs^12, 13^. Specifically, colon cancer chemotherapeutic drugs such as 5-Flurouracil and oxaliplatin include the risk of infections, changes in the heart rhythm, fatigue, anemia, and peripheral neuropathy^14^. Anthracycline drugs, such as doxorubicin (DOX), are most commonly used for the therapy of leukemia, lymphoma and breast cancer, but not for CRC. Although DOX has been shown to be a better adjuvant chemotherapy drug for CRC at advance stages^15^, the high concentration of the drug used in such cases can cause severe cardiotoxicity^16^. Several approaches including dose variations, use of derivatives of DOX, and treatment with adjuvants have been tried to decrease the cardiotoxicity associated with DOX^17^. However, neither alterations in the drug nor combinatorial therapy have been successful. Another major cause of DOX failure in CRC has been a progressive development of drug resistance^18^. Although the mechanism(s) that makes the CRC resistant to DOX is not clear, the increased expression of multi-drug resistance proteins, drug transporter proteins, and genetic/epigenetic changes mediated by DOX-induced increased ROS are thought to contribute to CRC resistance to chemotherapeutic drugs^19–21^. Furthermore, the sensitivity of CRC cells decreases not only to DOX but also to other types of anthracycline drugs such as daunorubicin, idarubicin, and epirubicin.

Our recent studies demonstrate that the polyol pathway enzyme, aldose reductase (AR; AKR1B1), plays an important role in oxidative stress-induced inflammatory signaling^22–24^. Inhibition of AR by pharmacological agents or genetic manipulations prevents the activation of various kinases (such as PKC, MAPK, ERK) that cause activation of transcription factors such as NF-κB and AP-1 that are known to transcribe various inflammatory and carcinogenic markers^10, 22, 25, 26^. We have shown that AR inhibition prevents colon cancer cells growth in tissue culture, nude mouse xenografts, and chemically-induced animal models of colon cancer. Most importantly, using nude mouse models, we have shown that AR inhibition halts colon cancer invasion, migration, and metastasis^27–32^. Furthermore, AR inhibition significantly inhibited angiogenesis, a major contributing factor to tumor growth and metastasis *in vivo* rodent models^33^. Based on these results, we investigated whether AR inhibitors could be used as adjuvant drug therapy along with chemotherapeutic drugs such as DOX to prevent CRC growth and metastasis.

In the present study, we have examined the effect of fidarestat, an AR inhibitor that has undergone a Phase -III clinical trial for the treatment of diabetic nephropathy and found to have no major side effects, in increasing the sensitivity of human CRC cells to DOX using cultured cancer cells as well as a nude mouse xenograft model. We found that fidarestat decreases growth of CRC cells and the expression of drug transporter proteins such as multidrug resistance protein 1 (MDR1 or Pgp-1 or ABCB1), multidrug resistance-associated protein 1 (MRP1 or ABCC1) and ATP-binding cassette subfamily G member 2 (ABCG2), which would increase the cytotoxic effects of DOX towards CRC cells. Fidarestat also limited DOX-induced cardiotoxicity and cardiac dysfunction in mice. Taken together, our results indicate a novel use of the AR inhibitor, fidarestat, as an adjuvant drug in combination with DOX to enhance antitumor efficacy for colon cancer and to decrease the DOX-induced cardiomyopathy effects in general.

## Results

### Fidarestat enhances DOX sensitivity in colon cancer cells

**Figure 1:**
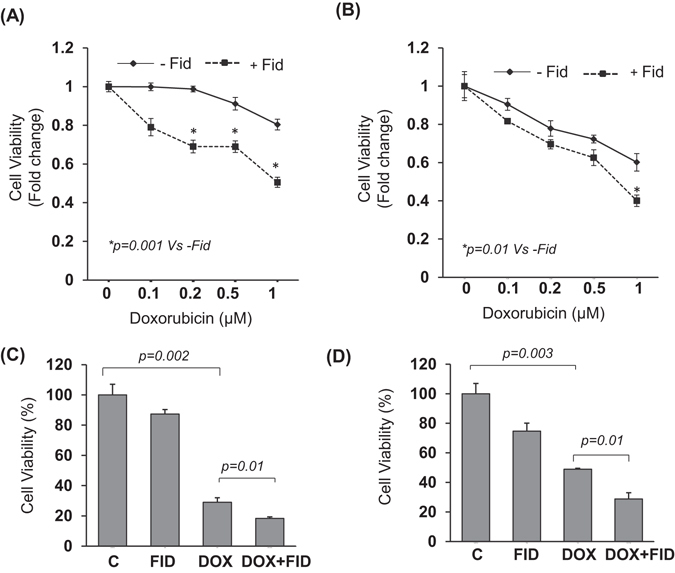
Inhibition of AR enhances the sensitivity of colon cancer cells to DOX: MTT cell viability assay showing the effect of chemotherapeutic drug DOX (0.1–1 μM) after 72 h of treatment without or with fidarestat (30 µM) in colon cancer cells (**A**) HT-29 and (**B**) SW480. Cell death was also assessed by Trypan blue cell exclusion assay after 72 h of treatment of (**C**) HT-29 and (**D**) SW480 CRC cells with DOX (1 μM) without or with fidarestat (30 µM). Values are Mean ± SD (n = 5). Individual *p* values are mentioned in the figures.

A dose-dependent increase in cell death was observed in colon cancer cells treated with DOX. The combination of DOX with fidarestat (30 µM) resulted in an additive effect on DOX-induced cell death. Approximately 25% increase in cell death was observed in colon cancer cells (HT-29 and SW480) treated with DOX in combination with fidarestat as compared to DOX alone (Fig. 1A and B). The increase in cell death induced by DOX in combination with fidarestat compared to DOX alone was further confirmed by Trypan blue exclusion assay (Fig. 1C and D). Similar results were observed when the death of HT-29 and SW480 cells was measured using AnnexinV/7-AAD staining (Supplementary Fig. 1A and B).

### Fidarestat enhances intracellular accumulation of DOX in colon cancer cells

**Figure 2:**
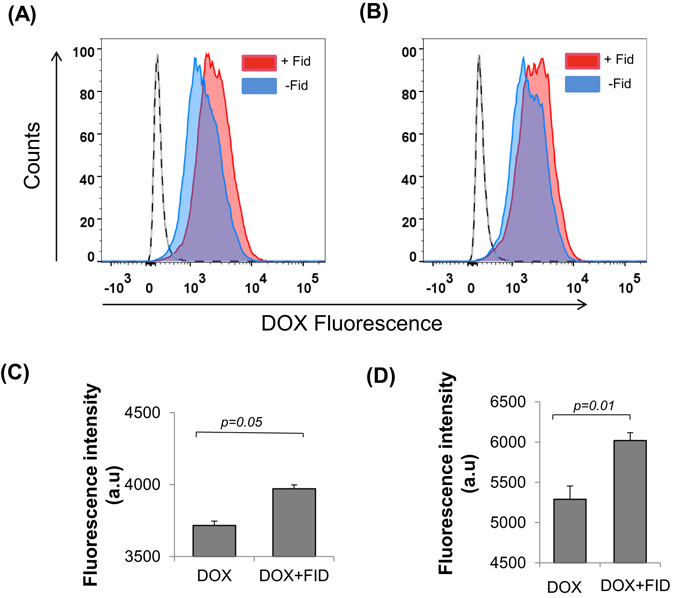
DOX accumulation in colon cancer cells: Flow cytometric analysis showing DOX fluorescence in (**A**) HT-29 and (**B**) SW480 colon cancer cells treated with DOX (1 µM) in the absence or presence of fidarestat (30 μM) for 24 h. Red filled histogram is DOX in combination with fidarestat and blue filled is DOX alone. Unfilled histogram with a solid line is untreated control and unfilled histogram with dashed line is fidarestat only treated (n = 3). Bar graph showing DOX (2 μM) fluorescence in (**C**) HT-29 and (**D**) SW480 cells treated alone or in combination with fidarestat (30 μM) for 6 h and analyzed by fluorescence spectrophotometry. Values are Mean ± SD (n = 3). Individual *p* values are mentioned in the figures.

As determined by flow cytometric analysis, higher intracellular accumulation of DOX was observed in colon cancer cells HT-29 and SW480 treated with DOX for 24 h in combination with fidarestat compared to DOX alone (Fig. 2A and B). The increase in DOX accumulation in colon cancer cells was also confirmed by measuring the fluorescence of HT-29 and SW480 cell lysates treated with DOX alone or in combination with fidarestat by fluorescence spectrophotometry (Fig. 2C and D).

### Fidarestat limits DOX-induced upregulation of drug transporters

**Figure 3:**
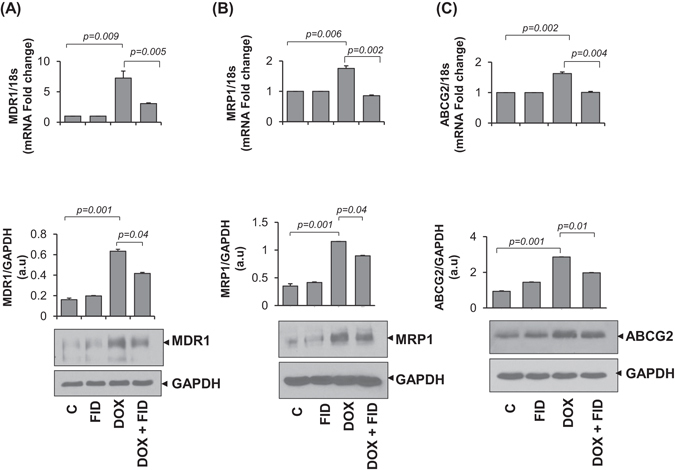
Expression of drug transporters in colon cancer cells: Gene and protein expression levels of drug transporters (**A**) MDR1 (**B**) MRP1 and (**C**) ABCG2 in HT-29 cells after 24 h of treatment with DOX (1 µM) without or with fidarestat (30 µM). Histograms showing quantification of blots along with *p* values mentioned in the figures. (n = 3). The representative cropped blots are shown and the full-length blots are presented in Supplementary Fig. 4.

Multidrug transporter proteins are involved in enhancing chemo-resistance of cancer cells by pumping the cytotoxic drugs out of the cells, thereby protecting the cells from the cytotoxic effects of the chemotherapeutic drugs. Exposure of HT-29 cells to DOX resulted in ~2 fold increase in the protein expression of the drug transporters such as MDR1, MRP1, and ABCG2 as well as their mRNA levels and the increase was significantly less in cells treated with DOX in combination with fidarestat (Fig. 3A,B and C). Similar results were observed in SW480 cells (Supplementary Fig. 2A,B and C). Thus, the decrease in the expression of drug transporters may explain the increased accumulation of DOX in CRC cells treated with DOX in combination with fidarestat compared to DOX alone.

### Fidarestat decreases the DOX-induced activation of NF-κB and phosphorylation of p38MAPK, ERK1/2 and SAPK/JNK

**Figure 4:**
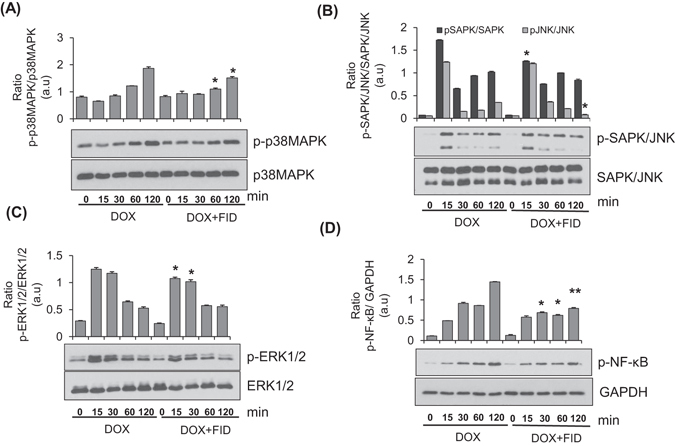
Effect of fidarestat on doxorubicin–induced activation of signaling pathways: Western blots showing the effect of fidarestat on DOX (1 μM)-induced activation of (**A**) p38MAPK, (**B**) SAPK/JNK (**C**) ERK1/2 and (**D**) NF-κB in SW480 cells treated alone or in combination with fidarestat (30 µM) (n = 3) for indicated time periods (0–120 min). The blots were quantified by Image J software and the histograms are shown. *p < 0.05, **p < 0.005 when compared to DOX-alone treated. The representative cropped blots are shown and the full-length blots are presented in Supplementary Fig. 5.

To examine how fidarestat decreases the expression of transporter proteins, we determined the activation of NF-κB and protein kinases such as p38 MAPK, ERK1/2 and SAPK/JNK in SW480 cells (Fig. 4A–D). Results shown in the Fig. 4D indicate that DOX caused a time-dependent increase in the phosphorylation of NF-κB and fidarestat pretreatment prevented it. Fidarestat also prevented the phosphorylation of p38MAPK, ERK1/2 and SAPK/JNK induced by DOX in colon cancer cells (Fig. 4A–C). These results suggest that by preventing the activation of NF-κB signals, AR inhibitor could prevent the expression of drug transporter proteins in CRC cells.

### Fidarestat potentiates DOX-induced inhibition of HT-29 colon tumor xenograft growth

**Figure 5:**
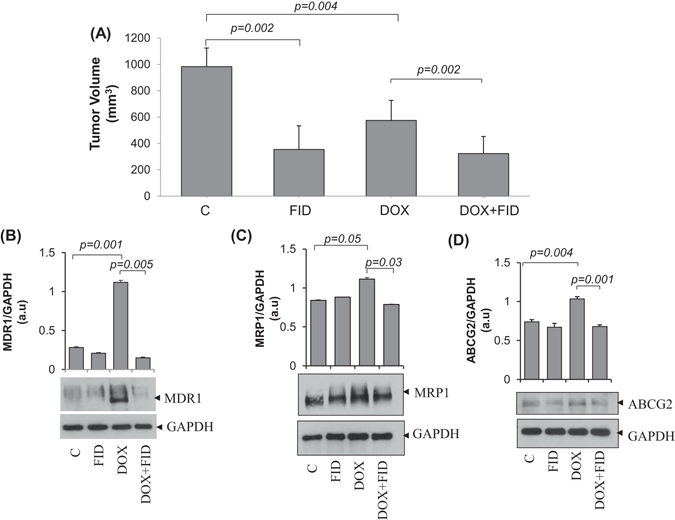
Effect of fidarestat in combination with doxorubicin *in vivo*: (**A**) Bars showing HT-29 xenograft tumor volume observed in nude mice after 21 days of treatment with DOX (4 mg kg^−1^ wk^−1^ i.p) alone or in combination with fidarestat in drinking water (25 mg kg^−1^). (n = 6 in each group). Western blots showing expression of drug transporters (**B**) MDR1 (**C**) MRP1 and (**D**) ABCG2 in HT-29 xenograft tumor samples. The representative cropped blots are shown and the full-length blots are presented in Supplementary Fig. 6. Bars showing densitometry analysis by Image J software. Values are Mean ± SD. (n = 3) Individual *p* values are mentioned in the figures.

We next confirmed our *in vitro* studies using nude mice xenografts injected with human CRC cells. A significant decrease in tumor volume was observed in mice treated with DOX (4 mg kg^−1^) in combination with fidarestat in the drinking water (25 mg kg^−1^) compared to DOX (4 mg kg^−1^ wk^−1^) alone after 21 days of treatment (Fig. 5A). Similar to the results observed in CRC cells, the expression of MDR1, MRP1 and ABCG2 was also increased in the DOX-treated xenograft tumors and the increase was significantly prevented by fidarestat (Fig. 5B,C,D). Further, a significant reduction in the levels of various cytokines such as endoglin, endothelin-1, FGF-1, FGF-2, follistatin, IL-8, PLGF and VEGF-A was observed in the homogenates of HT-29 tumor xenografts obtained from mice treated with DOX in combination with fidarestat, which indicates that AR inhibition prevents DOX-induced inflammatory cytokines and chemokines in the tumor tissues and thereby reduce tumor growth (Table 1).

**Table 1:** Inflammatory cytokines in HT-29 xenograft tumors.

	C	FID	DOX	DOX+FID
*Angiopoietin-2*	12.7 ± 1.6	13.2 ± 2.04	12.7 ± 2.1	15.2 ± 1.1
*BMP-9*	4.09 ± 0.5	4.3 ± 0.6	4.1 ± 0.3	4.03 ± 0.4
*EGF*	5.3 ± 1.9	6.1 ± 1.6	5.3 ± 1.9	7.2 ± 0.5
*Endoglin*	2347.09 ± 424.8	2215.9 ± 387.4	2322.1 ± 226.2*	1932.6 ± 266.7^#^
*Endothelin-1*	37.1 ± 8.5	36.5 ± 6.9	26.9 ± 4.3*	17.7 ± 2.4^#^
*FGF-1*	93.5 ± 12.1	106.3 ± 39.2	58.6 ± 29.2*	38.04 ± 10.8^#^
*FGF-2*	2342.06 ± 950.2	2065.5 ± 113.8	1728.5 ± 425.1*	1118.2 ± 402.9
*Follistatin*	114.1 ± 13.4	132.5 ± 26.8	168.01 ± 41.4*	135.4 ± 6.8
*HB-EGF*	38.51 ± 9.85	38.3 ± 4.7	34.5 ± 6.6	27.3 ± 3.08^#^
*HGF*	28.9 ± 14.3	24.6 ± 4.9	24.1 ± 8.09	22.5 ± 1.9
*IL-8*	1248.2 ± 423.01	1235.7 ± 344.1	2168.1 ± 596.6**	1459 ± 803.5^##^
*Leptin*	104.1 ± 16.2	105.6 ± 12.3	92.9 ± 29.06	104.9 ± 35.009^#^
*PLGF*	14.4 ± 4.4	10.9 ± 2.8	26.9 ± 9.4*	15.6 ± 5.06^##^
*VEGF-A*	3660.8 ± 542.8	3980.9 ± 596.5	4223.7 ± 407.9*	3670.1 ± 297.4
*VEGF-C*	7.1 ± 1.02	6.6 ± 1.08	5.5 ± 0.4	5.8 ± 0.7
*VEGF-D*	25.6 ± 3.3	26.7 ± 6.5	22.9 ± 1.04	22.01 ± 2.04

Expression of inflammatory cytokines in HT-29 xenograft tumors in nude mice after 21 days of treatment with DOX (4 mg kg^−1^ wk^−1^ i.p) alone or in combination with fidarestat in drinking water (25 mg kg^−1^). The tumors were homogenized in MILLIPLEX MAP Lysis Buffer and analyzed by using a human inflammatory cytokine/chemokine magnetic bead panel (Milliplex MAP Kit) using a Millipore Milliplex analyzer system. Values are Mean ± SD. Samples were analyzed in triplicates. (n = 6 in each group).

*p < 0.01 vs Control; **p < 0.001 vs Control; ^#^p < 0.01 vs DOX; ^##^p < 0.001 vs DOX.

### Fidarestat ameliorates DOX-induced cardiotoxicity

**Figure 6:**
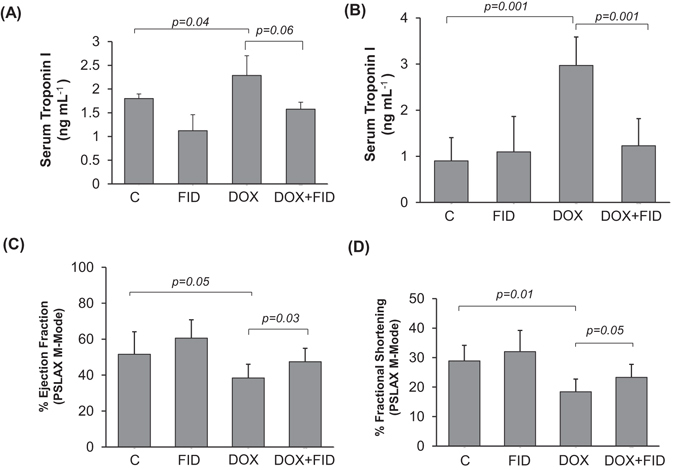
Fidarestat ameliorates DOX-induced cardiac dysfunction *in vivo*. Serum troponin I level in (**A**) tumor-bearing nude mice after 21 days of treatment with DOX (4 mg kg^−1^ wk^−1^ i.p) alone or with fidarestat (**B**) C57BL/6 J mice treated with DOX (10 mg kg^−1^ wk^−1^ i.p) alone or in combination with fidarestat (25 mg kg^−1^) after 10 days. Percentage Ejection fraction (**C**) and Fractional shortening (**D**) in heart of C57BL/6 J mice after 2-weeks of treatment with DOX (4 mg kg^−1^ wk^−1^ i.p) alone or in combination with fidarestat in drinking water (25 mg kg^−1^). Values are Mean ± SD. Individual *p* values are mentioned in the figures (n = 6 in each group).

DOX is well known to cause severe cardiotoxic side-effects *in vivo*. This limits the use of DOX in colon cancer and several other forms of cancer. Approximately 2-fold increase in the levels of troponin-I was observed in serum of tumor-bearing nude mice treated with DOX (4 mg kg^−1^), whereas in tumor-bearing nude mice treated with DOX in combination with fidarestat, the levels of troponin- I were similar to untreated mice, indicating cardio-protective functions of AR inhibitor (Fig. 6A). Apart from the reduction in serum troponin- I levels in mice treated with DOX in combination with fidarestat, decreased levels of pro-inflammatory cytokines like G-CSF, IL-1α, IL-1β, TNF-α, IL-6, IL-15, and IP-10 (Supplementary Table 1) were observed in the serum of tumor-bearing mice treated with DOX in combination with fidarestat compared to DOX alone, indicating anti-inflammatory role of fidarestat. Further, analyzing the levels of inflammatory cytokines and chemokines in the heart tissue of tumor-bearing nude mice showed similar results indicating anti-inflammatory effects of fidarestat on DOX-induced cardiotoxicity (Supplementary Table 2).

### Fidarestat limits DOX-induced cardiac dysfunction *in vivo*

**Figure 7:**
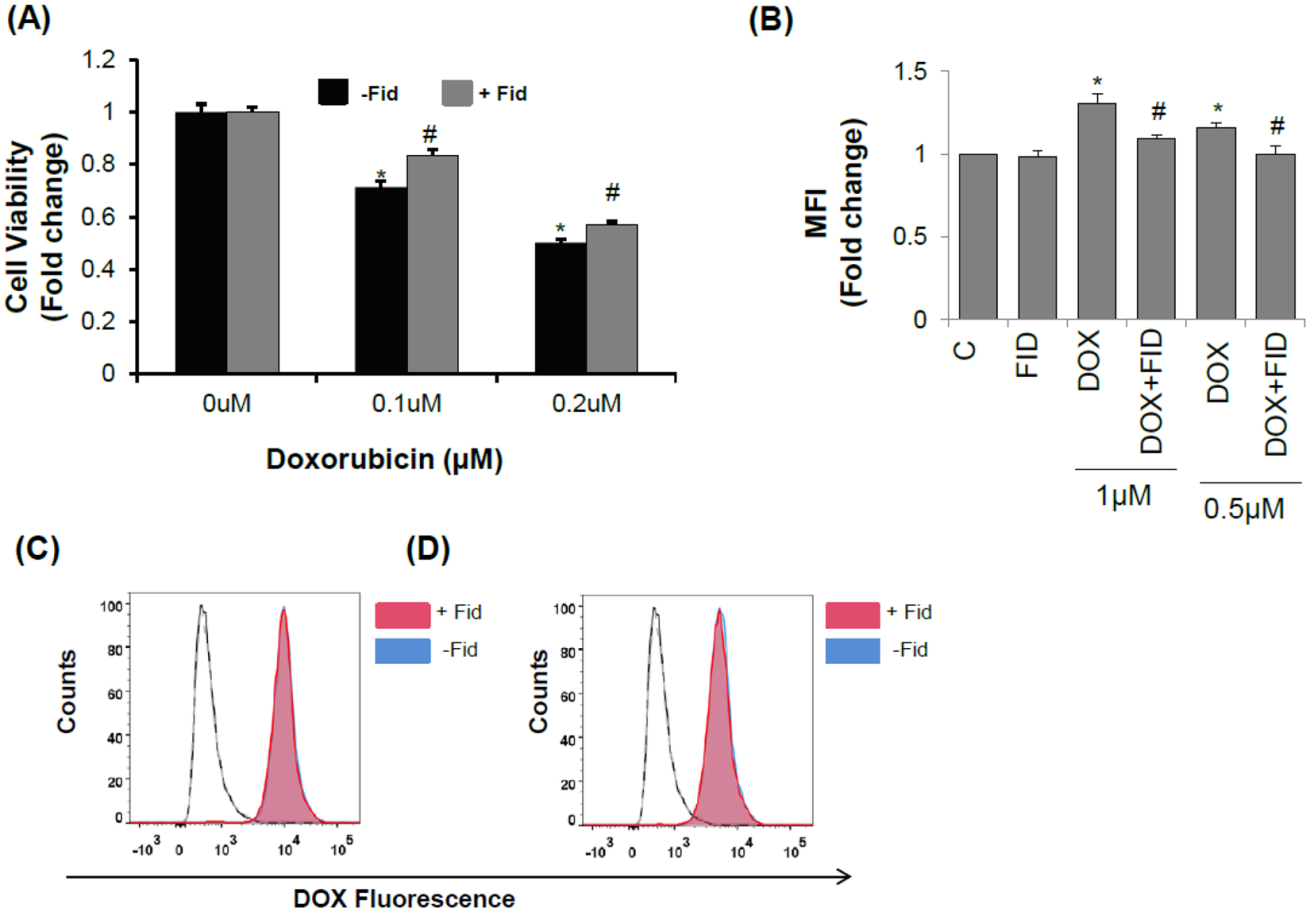
Effect of fidarestat on DOX-treated H9c2 cardiomyocytes: (**A**) Effect of DOX on H9c2 cell viability analyzed by MTT cell viability assay. Values are Mean ± SD (n = 6). (**B**) Effect of fidarestat on DOX-induced ROS production in H9c2 cells treated alone or in combination with fidarestat (n = 3). Doxorubicin accumulation in H9c2 cells treated with (**C**) 0.5 μM and (**D**) 1 μM DOX alone or in combination with fidarestat for 24 h. Red filled histogram is DOX in combination with fidarestat and blue filled is DOX alone. Unfilled histogram with a solid line is untreated control and unfilled histogram with dashed line is fidarestat only treated (n = 3). Individual *p* values are mentioned in the figures (n = 3).

To further investigate the cardio-protective effects of fidarestat, we next treated normal mice with DOX in the absence and presence of fidarestat and examined cardiac toxicity. An approximately 2.5-fold increase in the levels of troponin-I was observed in the serum of normal mice treated with DOX and the levels were significantly less in mice treated with DOX plus fidarestat, indicating cardio-protective functions of fidarestat against DOX (Fig. 6B). In addition to reduction in serum troponin-I levels in mice treated with DOX in combination with fidarestat, decreased levels of pro-inflammatory cytokines like G-CSF, TNF-α, IL-1α, IL-2, IL-3, IL-6, IL-15, and IP-10 were observed in the serum of C57BL/6J mice treated with DOX in combination with fidarestat (Table 2) indicating anti-inflammatory role of fidarestat as observed in the nude mice xenograft studies. We further, confirmed fidarestat’s cardio-protective effects by measuring the heart function by echocardiography. Similar to serum cardiotoxic biomarker, troponin-I, echocardiography also showed a significant decrease in ejection fraction (Fig. 6C) and fractional shortening (Fig. 6D) in C57BL/6J mice treated with DOX which was significantly restored in mice treated with DOX in combination with fidarestat. Further, fidarestat also decreased DOX-induced levels of malondialdehyde and protein-HNE adducts in the heart tissues suggesting that AR inhibitor prevents DOX-induced oxidative stress (Supplementary Fig. 3A and B). In addition, fidarestat prevented the DOX-induced decrease in the cell viability of normal H9c2 cardiac myocytes (Fig. 7A). Fidarestat also prevented the DOX-induced production of ROS in H9c2 cardiac myocytes (Fig. 7B). Interestingly, fidarestat did not change intracellular levels of DOX in H9c2 cells suggesting that in cancer cells, fidarestat decreases the efflux of DOX while in normal cells it doesn’t (Fig. 7C and D). Thus, our results demonstrate that fidarestat can ameliorate DOX-induced cardiac toxicity.

**Table 2:** Inflammatory cytokines in serum of C57BL/6J mice treated alone or in combination with fidarestat.

	C	FID	DOX	DOX+FID
*G-CSF*	201.7 ± 70.02	146.4 ± 35.1*	> 10000**	1325.3 ± 673.6^##^
*GM-CSF*	12.2 ± 3.3	36.7 ± 32.9	72.9 ± 13.6*	34.1 ± 18.5^##^
*IFNγ*	ND	ND	31.1 ± 20.3*	7.1 ± 5.1^##^
*IL-1α*	128.2 ± 51.6	89.3 ± 31.7*	284.7 ± 105.6*	185.7 ± 45.1^#^
*IL-1β*	4.9 ± 3.6	5.3 ± 1.3	73.9 ± 35.8**	69.2 ± 36.3
*IL-2*	ND	ND	12.3 ± 6.9*	3.1 ± 0.2^##^
*IL-3*	ND	31.1 ± 11.06	490.4 ± 215.2*	213.7 ± 184.2^##^
*IL-6*	ND	ND	>10000*	1650.5 ± 118.1
*IL-7*	ND	ND	23.7 ± 10.5	17.6 ± 13.6^#^
*IL-9*	42.9 ± 8.2	71.3 ± 5.9*	371.9 ± 139.1**	113.8 ± 51.5^##^
*IL-10*	8.1 ± 3.7	16.3 ± 8.3*	70.6 ± 24.7**	36.9 ± 19.2^#^
*IL-12* (*p40*)	19.3 ± 14.1	23.3 ± 11.3	63.3 ± 22.4**	24.1 ± 4.7^##^
*IL-12* (*p70*)	20.8 ± 14.1	14.9 ± 3.6	143.1 ± 57.7**	127.5 ± 61.04
*IL-13*	156.1 ± 27.01	131.7 ± 58.9	1382.8 ± 699.01**	231.9 ± 20.8^##^
*IL-15*	35.9 ± 13.2	50.1 ± 22.6	149.1 ± 73.7**	68.9 ± 19.7^##^
*IL-17*	0.3 ± 0.1	1.1 ± 0.5	6.8 ± 3.6**	7.4 ± 2.5
*IP-10*	192.7 ± 32.3	216.2 ± 140.1	973.5 ± 366.1**	375.1 ± 34.7^##^
*KC*	42.8 ± 17.4	69.1 ± 15.05	286.7 ± 75.4**	187.6 ± 132.5^#^
*MCP-1*	21.2 ± 6.4	17.01 ± 6.9	199.2 ± 86.8**	82.8 ± 43.7^##^
*MIP-1α*	21.6 ± 10.1	23.3 ± 6.06	151.05 ± 95.8**	28.02 ± 9.1^##^
*MIP-1β*	13.1 ± 5.4	11.6 ± 3.04	280.2 ± 134.7**	24.1 ± 13.6^##^
*MIP-2*	53.8 ± 5.8	49.02 ± 5.4	131.1 ± 17.6**	54.9 ± 10.7^##^
*RANTES*	10.1 ± 2.9	5.8 ± 1.9	29.3 ± 17.6**	12.6 ± 4.9^##^
*TNFα*	ND	ND	21.10 ± 18.7**	6.1 ± 1.7^##^

Serum cytokines measured in normal mice after 10days of treatment with DOX (10 mg kg^−1^ wk^−1^ i.p) alone or with fidarestat in drinking water (25 mg kg^−1^). Blood was collected from individual mouse and serum samples were analyzed by using a mouse inflammatory cytokine/chemokine magnetic bead panel (Milliplex MAP Kit) using a Millipore Milliplex analyzer system. Samples were analyzed in triplicates. Values are Mean ± SD (n = 6 in each group).

*p < 0.01 vs Control; **p < 0.001 vs Control; ^#^p < 0.01 vs DOX; ^##^p ^<^ 0.001 vs DOX; ND = not detectable.

## Discussion

Chemotherapy, a major therapeutic approach to treat solid tumors prior or subsequent to surgery, has been shown to improve the tumor diminution and survival of CRC patients. Anthracyclines, specifically DOX are most commonly used in clinical practice to treat solid tumors of breast and lung cancers. However, DOX is not usually used in CRC because its efficacy is often reduced due to the development of drug resistance, leading to a significantly higher dose of drug required to treat CRC. Since DOX is cardiotoxic, higher amounts of this class of potent drugs limit its use in CRC therapy^34^. Therefore, development of novel therapeutic approaches that could reverse drug resistance as well as prevent cardiotoxicity is required^35, 36^. In the present study, for the first time, we have demonstrated that the AR inhibitor, fidarestat, increases the sensitivity of CRC to DOX and decreases the cardiomyopathy associated with DOX *in vitro* and *in vivo*.

We have demonstrated earlier that the AR inhibitors prevent colon cancer growth and metastasis by inhibiting the NF-κB-mediated inflammatory signaling and VEGF-induced neo-vascularization^27, 28, 30, 32^. Further, our studies also suggest that AR inhibitors such as fidarestat, zopolrestat, and sorbinil are tumoristatic but not tumoricidal^28, 30, 37^. Since adjuvant effects of AR in combination with chemotherapeutic drugs are not known, we examined the efficacy of the combination of AR inhibitors with chemotherapeutic drugs in chemotherapy and decrease in the unwanted side effects. Our current data shown in the Fig. 1 strikingly indicate that combination of DOX with AR inhibitor fidarestat significantly enhances the sensitivity of colon cancer cells HT-29 and SW480 to DOX. A 30–40% increase in cell death was observed in colon cancer cells treated with DOX in combination with fidarestat compared to DOX alone, suggesting that fidarestat increases the sensitivity of CRC to anthracyclines. One of the major problems encountered during chemotherapy is the progressive decrease in sensitivity to chemotherapeutic drugs. The activity of drug transporters of the ATP-binding cassette (ABC) family such as MDR1 (Pgp-1), the ABCC family such as MRP1 and MRP2, breast cancer resistant protein family (BCRP) such as ABCG2 etc. in cancer cells leads to decrease in the intracellular concentration of the chemotherapeutic drugs by ATP-dependent efflux of unmodified drug from the cells, which contribute to drug-resistance^38–41^. Our results indicate that intracellular DOX accumulation in CRC is increased in CRC cells treated with DOX in combination with fidarestat when compared with DOX alone. Next, we examined if fidarestat increased the efficacy of DOX sensitivity towards colon cancer by altering the expression of drug transporters. Our results demonstrated that the levels of drug transporter proteins such as MDR1, MRP1, and ABCG2 were up-regulated in colon cancer cells treated with DOX and fidarestat treatment prevented the DOX-induced increase in the MDR1, MRP1, and ABCG2. DOX alone or in combination with fidarestat did not affect the levels of other transporter proteins such as RLIP76 and MRP2 (data not shown). Thus, by decreasing the specific expression of MDR1, MRP1, and ABCG2, fidarestat prevents the efflux of DOX and enhances the cytotoxic effects. The *in vitro* observations were further confirmed by *in vivo* studies, wherein, we observed a higher percentage of tumor reduction in HT-29 xenograft tumors treated with DOX in combination with fidarestat as compared to DOX alone. Consistent with our results, several antioxidants have been shown to decrease the expression of multidrug resistance conferring proteins such as P-glycoprotein (MDR1), MRP1 and ABCG2 and increase the sensitivity of chemotherapeutic agents not only towards colon cancer but also for lung and breast cancers^39, 42–44^. Since the clinical use of DOX is hampered mainly due to its associated cardio-toxic side effects, we next examined the role of AR inhibitor in reducing the DOX-induced cardiotoxicity in *in-vitro* and *in vivo* models. Our data shown in the Fig. 7A indicate that DOX-induced decrease in the H9c2 cardiac myocytes viability is prevented by pretreatment with fidarestat.

The DOX-associated cardiotoxicity is either attributed to the production of reactive oxygen species (ROS)^45, 46^ or due to the reductive metabolism of DOX to doxorubicinol, which is more potent cardiotoxic compound compared to the parent compound DOX^47–49^. Our studies using H9c2 cells suggest that fidarestat prevents DOX–induced ROS generation as well as the formation of protein-HNE adducts, a marker of increased oxidative stress. It is possible that by decreasing the oxidative stress-induced formation of ROS, AR inhibitor prevents DOX–induced cardiac toxicity. Further, AR is among the class of Phase I drug-metabolizing enzymes like Carbonyl Reductases (CBR) and has been reported to have high substrate specificity for anthracycline drugs such as DOX and daunorubicin and reduce the parent compounds to their respective alcohol derivatives^48, 50–55^. Therefore, it is likely that inhibition of AR could prevent DOX-induced cardiotoxicity by preventing the formation of cardiotoxic doxorubicinol. Consistent with our results, few reports also suggest that increase in aldo-keto reductases (AKRs) such as AKR1A1, AKR1B10, AKR1C1, AKR1C2, AKR1C3, AKR1C4 have been shown to alter the metabolism of anthracyclines and acquired drug resistance^50, 51, 54^. AKRs such as AKR1C2 and AKR1C3 have been shown to induce DOX resistance by catalyzing the conversion of DOX to the less toxic doxorubicinol. Further, AKR1B10, besides participating in the DOX metabolism, has also been shown to be responsible for drug resistance in gastric and lung cancer cells^50, 51^. Furthermore, serum and cardiac troponin levels are useful indicators of acute cardiomyocyte injury in many clinical settings like heart failure, pulmonary embolism, stroke, sepsis and drug-induced cardiotoxicity^56^. Specifically, cardiac troponin-I (TnI) and cardiac troponin-T (TnT) are the two sensitive biomarkers of cardiac damage and important indicators of detecting acute myocardial infarctions^57, 58^. Our data shown in the Fig. 6A indicate that the tumor-bearing nude mice treated with DOX showed an approximately 2-fold increase in the serum troponin-I levels compared to control mice. However, no increase was observed in mice treated with DOX in combination with fidarestat suggesting that fidarestat prevents DOX–induced cardio-toxicity. The cardio-protective functions of fidarestat in DOX-induced cardiotoxicity were further confirmed by another set of experiments using a normal C57BL/6J mice model. There was ~2.5-fold increase in the levels of troponin-I in the serum of DOX-treated mice heart compared to DOX plus fidarestat-treated mice (Fig. 6B). Further, our echocardiography studies shown in the Fig. 6C and D suggest a significant decrease in the ventricular ejection fraction and fractional shortening in DOX-treated mice which was restored by fidarestat.

Another major cause of DOX –induced cardiotoxicity is increased oxidative stress, which induces secretion of various inflammatory cytokines and chemokines that contribute to poor prognosis and complicate the symptoms associated with the pathologies where DOX is used as treatment^59–63^. Elevated levels of pro-inflammatory cytokines have been reported in the vasculature and myocardium during DOX-induced cardiotoxicity^64–66^. Analyzing the cytokine levels in serum of mice treated with DOX showed a significant increase in the levels of pro-inflammatory cytokines such as interleukins, TNF-α and GM-CSF, whereas in mice treated with DOX in combination with fidarestat prevented it. The decrease in the expression of inflammatory markers may also contribute to a reduction in cardiotoxicity observed in mice treated with DOX in combination with fidarestat compared to DOX alone.

In conclusion, the lack of effective therapies for advanced CRC relates to the current poor understanding of the progression of the disease, especially the processes leading to metastasis which is often incurable and leads to patient’s death. Moreover, currently available drugs such as oxaliplatin, 5-FU, and irinotecan are very expensive and cause severe toxicities^16^. Therefore, new strategies are required for the prevention and therapy of CRC and metastasis. We propose that a combination of chemotherapeutic drugs and AR inhibitors could improve the sensitivity of chemo-drugs towards cancer cell death and decrease the amount of anti-carcinogenic drugs thereby decrease the cardiotoxicity associated with chemotherapeutic drugs. Although DOX is a very effective and relatively inexpensive drug for the chemotherapy of different types of human cancers, its use in colon cancer is hitherto limited, mainly due to associated side effects such as cardiotoxicity and acquired drug resistance. Our results indicate that by decreasing the MDR1, MRP1, and ABCG2 drug transporters, AR inhibitor fidarestat increases the sensitivity of DOX to colon cancer by decreasing the efflux of drugs from the cells. Further, by decreasing the increased oxidative stress and inflammatory cytokines and chemokines, fidarestat prevents cardiomyopathy associated with DOX. Fidarestat is a water-soluble and most potent specific small molecule inhibitor of AR with an IC_50_ value of 9 nM^67, 68^. It can rapidly distribute into the tissues and binds to the AR protein selectively with high specificity thereby limiting any off-target effects. It has no apparent direct pharmacological effects other than AR inhibition and does not have an effect on hepatic drug-metabolizing enzymes. Further, fidarestat has been shown to be safe for human use without any irreversible toxicities in 52 weeks of Phase III clinical trials for diabetic neuropathy^67, 69^. Thus, our studies provide evidence that a potent and clinically safe AR inhibitor, fidarestat, could be a novel adjuvant drug to increase the sensitivity of colon cancer cells to DOX and also to prevent cardiotoxicity associated with the effective and relatively inexpensive synthetic anthracycline drugs.

## Materials and Methods

### Materials

McCoy’s 5A medium (#16600–082), RPMI-1640 (#11875–093), DMEM (#11965–092), Penicillin-Streptomycin (#15140), Trypsin-EDTA (#25200–056) were obtained from Invitrogen (Life Technologies). Dulbecco’s Phosphate Buffered Saline (PBS) (#21–030-CV) was obtained from Corning Cellgro. Fetal Bovine Serum (FBS) (#100-602) was obtained from Gemini Biosciences. 3-(4,5-Dimethyl-2-thiazolyl)-2,5-diphenyl-2H-tetrazolium bromide (MTT) (#M2128) and Doxorubicin Hydrochloride (DOX; #D1515 and #44583 for fluorescence studies) was obtained from Sigma Aldrich. Antibodies against MDR1 (D3H1Q), MRP1 (D708N), ABCG2 (D5V2K), phospho-p38MAPK (D3F9), p38MAPK (D13E1), phospho-p44/42 MAPK (Erk1/2) (D13.14.4E), p44/42 MAPK (Erk1/2) (137F5), phospho-SAPK-JNK (81E11), SAPK-JNK (9252) and GAPDH (14C10) were obtained from Cell Signaling Technology. Anti-4-Hydroxynonenal antibodies were obtained from Abcam (#ab46545). Anti-rabbit secondary antibodies (#170–6515) and protein standard for SDS-PAGE (#161–0375) were obtained from Bio-Rad. Annexin V-Alexa Fluor 488 (#A13201) and 7-aminoactinomycin D (7-AAD) (#A1310) were obtained from Molecular Probes, Invitrogen. Fidarestat was obtained from Livwel Therapeutics Inc, CA. USA.

### Cell Culture

Human colon cancer cell lines HT-29 (HTB-38), SW480 (CCL-228) and rat H9c2 cardiomyocytes (CRL-1446) were obtained from American Type Culture Collection (ATCC). HT-29 cells were maintained in McCoy’s 5A medium supplemented with 10% FBS and 1% penicillin-streptomycin. SW480 cell lines were maintained in RPMI-1640 supplemented with 10% FBS and 1% penicillin-streptomycin. Rat H9c2 cardiomyocytes were maintained in DMEM supplemented with 10% FBS and 1% penicillin-streptomycin. All the cell lines were obtained from ATCC and tested negative for mycoplasma, bacteria, yeast, and fungi. We followed universal precautions to handle all cell cultures. All the cells were maintained at 37 °C in a humidified atmosphere with 5% CO_2_.

### Cytotoxicity studies

Cells were plated at a density of 2000 cells/well in 96-well plates (n = 6 for each condition) and allowed to adhere overnight. For wells to be treated with fidarestat in combination with DOX, pre-treatment was done with fidarestat (30 µM) overnight. The cells were then treated with various concentrations of DOX (0.1–1 µM) for 72 h without or with fidarestat (30 µM). Cell viability at the end of incubation period was determined by MTT assay^29, 32^. Briefly, at the end of the incubation period, 10 μL of 5 mg/mL MTT was added to each well of the 96-well plate and incubated for 2 h. After 2 h, the supernatant was removed and the formazan crystals were dissolved in 100 μL DMSO and the absorbance was recorded at 570 nm using a BioTek Synergy-2 plate reader. The data is represented as fold change compared to untreated control. Cell viability was also assessed by Trypan Blue cell exclusion assay after 72 h of treatment with 1 μM DOX. Attached cells and the supernatant containing floating dead cells were collected from all the wells (n = 3) and stained with Trypan blue dye and the live cells were counted using a hemocytometer.

### Annexin V/7-AAD Staining

HT-29 and SW480 cells were seeded at a density of 30,000cells/cm^2^ in 6-well plates and treated with DOX alone or in combination with fidarestat (30 μM) for 48 h. After treatment, cells were harvested and stained with Annexin V and 7-AAD and incubated on ice for 30 min. The cells were then analyzed using a BD FACS Fortessa Flow Cytometer using a 488 nm laser and detection wavelengths of 530 nm (Alexa Fluor 488) and 670 nm (7-AAD). Data was analyzed using Flow Jo software (Treestar, OR, USA) and gates were drawn using untreated controls.

### Intracellular drug accumulation assay

Cells were grown in 60mm tissue culture dishes at a density of 50,000 cells/cm^2^. Pre-treatment was done with 30 µM fidarestat overnight for wells to be treated with fidarestat in combination with DOX. The cells were then treated with DOX (1 µM) without or with fidarestat (30 µM). After incubation with DOX alone or in combination with fidarestat for 24 h, the cells were washed with PBS, detached with Trypsin-EDTA and analyzed by a BD FACS Fortessa Flow Cytometer at UTMB flow cytometry core facility (Ex/Em: 470/585). Data was analyzed and the histograms were generated using Flow Jo Software (Treestar, Ashland, OR, USA). Intracellular DOX accumulation was also analyzed by fluorescence spectrophotometry using a BioTek Synergy-2 Plate Reader^70^. Briefly, after incubating the cells for 1 h with the indicated concentration of DOX without or with fidarestat (30 µM), the cells were washed with ice-cold PBS and detached with Trypsin-EDTA. Cells were then pelleted at 2100 g for a 30 seconds pulse spin and re-suspended in 500 µl of 1:1 mixture of ethanol and hydrochloric acid (HCl) (absolute ethanol: 0.3N HCl) and the fluorescence was measured with a BioTek Synergy-2 Plate Reader (Ex/Em: 470/585). Cell suspensions from untreated controls and fidarestat only treated were used to subtract the background fluorescence A small fraction was lysed with a sonicator and total protein content was analyzed by Bio-Rad Protein Assay Dye (Catalog#500–0006). Fluorescence values obtained were normalized to total protein content and represented as arbitrary fluorescence units in DOX and DOX + FID treated cells.

### Analysis of MDA levels in heart tissue homogenate of mouse

Lipid peroxidation marker, MDA, levels were measured in heart tissue homogenates of the mice using a kit (#21044) from Oxis International Inc., following manufacturer’s instructions. Briefly, heart tissue was homogenized in PBS containing 5 mM butylated hydroxytoluene (BHT). The lysate was cleared by centrifugation at 3000 × g for 10 min at 4 °C. The supernatant was used in the assay as per instructions provided with the kit and the absorbance was recorded at 586 nm using a plate reader. Total MDA levels (μM) were calculated based on the standard curve generated with the standards provided with the kit and normalized to protein levels. The values are represented as μMoles MDA μg^−1^ of protein. Protein-HNE conjugates were determined in the heart homogenates by Western blot analysis.

### Measurement of ROS accumulation in H9c2 cardiomyocytes

Intracellular ROS was measured by flow cytometry using CM-H2DCFDA (#C6827; Molecular Probes, Invitrogen). To analyze ROS levels, H9c2 cells were seeded in 6 well tissue culture dishes at a density of 20,000 cells/cm^2^. For cells to be treated with DOX in combination with fidarestat, pretreatment was done with 30 μM fidarestat overnight followed by treatment with 0.5 μM or 1 μM DOX for 1–2 h. After the incubation period, the cells were stained with CM-H2DCFDA for 15 min, harvested, and analyzed immediately by a Flow Cytometer (BD LSRII Fortessa). Data analysis was performed using Flow Jo (Treestar, OR, USA) and represented as fold change of Mean Fluorescence Intensity (MFI) compared to untreated control.

### Analysis of inflammatory cytokines in serum and tissue samples

Analysis of inflammatory cytokines in serum obtained from blood collected from treated animals was performed by using a mouse cytokine/chemokine magnetic bead multiplex kit from Millipore (#MCYTOMAG-70K) following manufacturer’s instructions. Briefly, blood collected from mice was allowed to clot for 30 min and centrifuged for 10 min at 1000 g. Serum supernatant was collected, diluted 1:2 in Milliplex assay buffer and incubated with the pre-mixed beads provided with the kit with agitation on a plate shaker overnight at 2–8 °C. After overnight incubation, wells were washed with wash buffer using an automated magnetic plate washer (ELx405; Biotek) and incubated with detection antibodies for 1 h at room temperature with agitation on a plate shaker. The wells containing detection antibodies were counterstained with Streptavidin-Phycoerythrin, incubated for another 30 min and washed again with a plate washer. After washing, 150 μL of sheath fluid was added to the wells and analyzed with a Luminex analyzer from Millipore. The results are expressed as pg mL^−1^ based on the standard curve generated with the standards provided with the kit using Luminex *x*PONENT software. For tissue sample analysis by Milliplex kit, fresh tissue sections were homogenized in MILLIPLEX MAP Lysis Buffer (#43–040) containing phosphatase and protease inhibitors. The homogenates were cleared by centrifugation and protein content was quantified with Bio-Rad Protein Assay Dye (Catalog#500–0006). 25 μg of protein in triplicates were used for each sample and incubated with magnetic beads from mouse cytokine and chemokine kit (#MCYTOMAG-70K) or human angiogenesis/growth factor magnetic bead panel (#HAGP1MAG-12K) and analyzed as mentioned above. Serum troponin-I levels were determined by an ELISA kit from Life Diagnostics (#CTNI-1-US).

### Western Blot analysis

Cells were washed twice with ice-cold PBS, harvested by scraping and whole cell lysates were prepared in RIPA buffer containing phosphatase and protease inhibitors (#SC-24949; Santa Cruz Biotechnologies). Cell lysates were incubated on ice for 30 min and clarified by centrifugation at 20,000 g for 30 min. Protein content was estimated using Bio-Rad protein Assay Dye (Catalog#500–0006). Equal amounts of protein (40 μg) were resolved in 12% SDS-PAGE gels, blotted onto PVDF membranes (#IPVH00010; EMD Millipore) and probed with antibodies for MDR1(D3H1Q; 1:1000; Cell Signaling), MRP1(D708N; 1:1000; Cell Signaling), ABCG2 (D5V2K; 1:1000; Cell Signaling), phospho-p38MAPK (D3F9; 1:1000; Cell Signaling), p38MAPK (D13E1; 1:1000; Cell Signaling), phospho-p44/42 MAPK (Erk1/2) (D13.14.4E; 1:1000; Cell Signaling), p44/42 MAPK (Erk1/2) (137F5; 1:1000; Cell Signaling) phospho-SAPK-JNK (81E11;1:1000; Cell Signaling), SAPK-JNK (#9252; 1:1000; Cell Signaling) and GAPDH (14C10; 1:1000; Cell Signaling). Secondary HRP-conjugated anti-rabbit antibody (#170–6515; 1:1000; Bio-Rad) was used. The antigen-antibody complexes were detected by chemiluminescence using Super Signal West Pico Chemiluminescent Substrate (#34080; Thermo Scientific, USA). Densitometry was performed using Image J software (NIH) and histograms showing relative expression of MDR1, MRP1 and ABCG2 normalized to GAPDH were generated.

### Semi-Quantitative Real Time PCR

All the gene expression analyses were performed at the UTMB Molecular Genomics core facility. RNA samples for Real-Time Analysis were quantified using a Nanodrop Spectrophotometer (Nanodrop Technologies) followed by analysis on an RNA Nano chip using an Agilent 2100 Bioanalyzer (Agilent Technologies). Synthesis of cDNA was performed with 0.5 μg or 1 μg of total RNA in a 20 μl reaction using the reagents in the Taqman Reverse Transcription Reagents Kit from Life Technologies (#N8080234). Q-PCR amplifications (performed in duplicate or triplicate) were done using 1 µl of cDNA in a total volume of 20 µl using the iTaq Universal SYBR Green Supermix (Bio-Rad #1725125). The final concentration of the primers was 300 nM. Relative RT-QPCR assays were performed with 18 s RNA as the internal house-keeping control. The relative transcriptional expression levels of the gene of interest (GOI) was calculated by normalizing the Ct values of the GOI with the average Ct values of 18 s RNA as ΔCt, the relative transcriptional expression of the GOI was calculated with 2^(−ΔΔCt)^.

The primer sequences used in the present study were: MDR1, *Forward*: 5′-GAGAGATCCTCACCAAGCGG-3′ *Reverse*: 5′-ATCATTGGCGAGCCTGGTAG-3′; MRP1, *Forward*: 5′-GAGAGATCCTCACCAAGCGG-3′ *Reverse*: 5′-ATCATTGGCGAGCCTGGTAG-3′; ABCG1 *Forward*: 5′-CTTCTTCCTGACGACCAACCAG-3′ *Reverse*: 5′-TCTGTAGTATCCGCTGATGTATTCATG-3′

All PCR assays were run on the ABI Prism 7500 Sequence Detection System and the conditions were: 50 °C, 2 min; 95 °C, 10 min (40 cycles); 95 °C, 15 seconds; 60 °C, 1 min.

### Nude mouse xenografts

Sub-confluent HT-29 CRC cells used for xenograft experiments were trypsinized and re-suspended in 100 µl (1 × 10^6^ cells/100 µl) of serum-free McCoy’s 5A media and injected subcutaneously into 6–8 weeks old male athymic nude mice (nu/nu) (Envigo). When the transplanted tumor’s mean diameter reached ~8 mm, the mice were randomly divided into 4 groups (n = 6 in each group): (a) Control, (b) fidarestat alone (25 mg kg^−1^) in drinking water, (c) DOX alone (4 mg kg^−1^ i.p) and (d) DOX plus fidarestat. DOX (4 mg kg^−1^) was administered intraperitoneally once a week for 3-weeks. The tumor volume and body weight were measured after every 3 days. The animals were euthanized when the mean tumor diameter reached ~20 mm. All animal experiments were carried out in accordance with a protocol approved by the Institutional Animal Care and Use Committee.

### Cardiac Function Assessment by Echocardiography

Echocardiography was performed on 4 groups (n = 6 in each group) of male mice (C57BL/6J, 5–6 weeks) to assess cardiac function: (1) control mice (no treatment); (2) mice given fidarestat orally in drinking water (25 mg kg^−1^) (3) Mice treated with DOX (4 mg kg^−1^ i.p) and (4) Mice treated with DOX (4 mg kg^−1^ i.p) and fidarestat orally (25 mg kg^−1^). Mice were anesthetized with a mixture of 1.5% Isoflurane and 95% Oxygen and recordings taken with a 30 MHz probe. The heart was imaged in B-mode and M-mode to examine the parameters of the left ventricle (LV) in diastole (−d) and systole (−s)^71, 72^ using a Vevo 2100 Analyzer (Visual Sonics, Toronto, Canada) at the UTMB Centre for Biomedical Imaging Core.

Fractional shortening percentage (FS) (%), a surrogate of systolic function, was calculated from left ventricular dimensions as follows:

1 $${\rm{FS}}( \% )=\{({\rm{LVED}}-{\rm{LVES}})/({\rm{LVED}})\}\times 100$$

LVED indicates left ventricular end-diastolic diameter and LVES is left ventricular end-systolic diameter and Ejection Fraction (EF) was calculated as follows:

2 $${\rm{EF}}( \% )=\{({\rm{Stroke}}\,{\rm{Volume}})/\mathrm{End}\,{\rm{Diastolic}}\,{\rm{Volume}})\}\times 100$$

All measurements were performed in triplicate and acquired in long-axis and short-axis views. Data were analyzed by using Vevo 2100 standard measurement software.

### Guideline statement

All methods used in this study are in accordance with the guidelines and regulations approved by UTMB, Galveston. All animal experiments were performed in accordance with relevant guidelines and protocols approved by Institutional Animal Care and Use Committee (IACUC), UTMB, Galveston.

### Data Availability

The data supporting the findings of this study are available within the article and its Supplementary Files. All other relevant source data are available from the corresponding authors upon request.

### Statistical Analysis

Data are presented as Mean ± SD or SEM and the *p* values were determined by Student *t* test for pair-wise comparisons and one-way ANNOVA was used for multiple comparisons using GraphPad Prism software. *p* < 0.05 was considered statistically significant.

## Electronic supplementary material

Supplementary figures
