# Supplementary material for: Aldose reductase inhibitor increases doxorubicin-sensitivity of colon cancer cells and decreases cardiotoxicity
Source: Sci Rep. 2017 Jun 9;7:3182. doi: 10.1038/s41598-017-03284-w (PMC5466629; doi:10.1038/s41598-017-03284-w)
Supplement: Supplementary file 1 — Supplementary figures [file 41598_2017_3284_MOESM1_ESM.pdf]

Supplementary info

Aldose reductase inhibitor increases doxorubicin-sensitivity of colon cancer cells and decreases cardiotoxicity.

Himangshu Sonowal, Pabitra B Pal, Jian-Jun Wen, Sanjay Awasthi, Kota V Ramana, Satish K Srivastava.

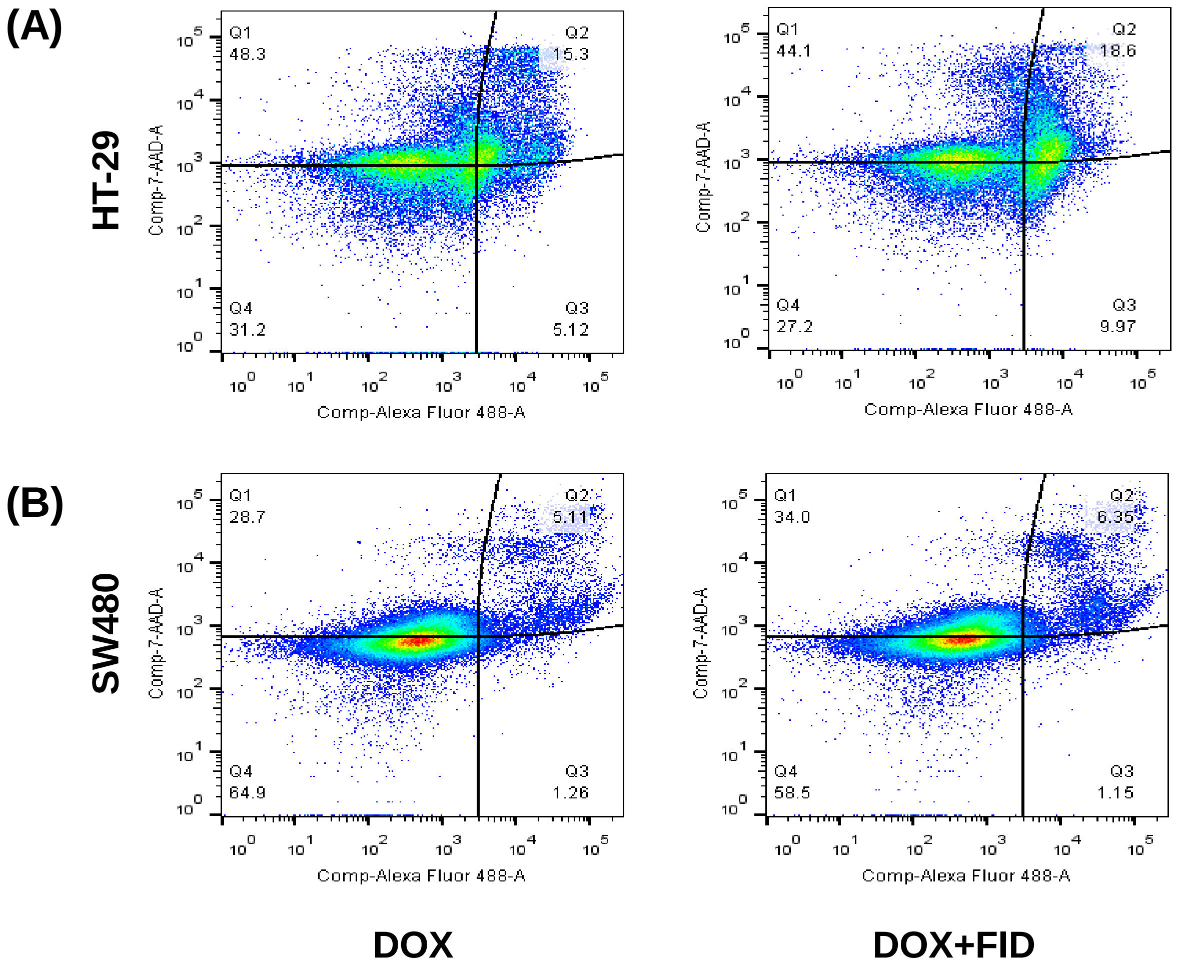

Supplementary Figure.1: Cell death in HT-29 and SW480 cells treated with DOX in combination with fidarestat: Annexin V/7-AAD staining analyzed by flow cytometry in (A) HT-29 and (B) SW480 cells treated with DOX alone or in combination with fidarestat for 48h. Data acquisition was done using a BD LSRII Fortessa and analyzed by Flow Jo software. Gates were plotted based on untreated control (n=3)

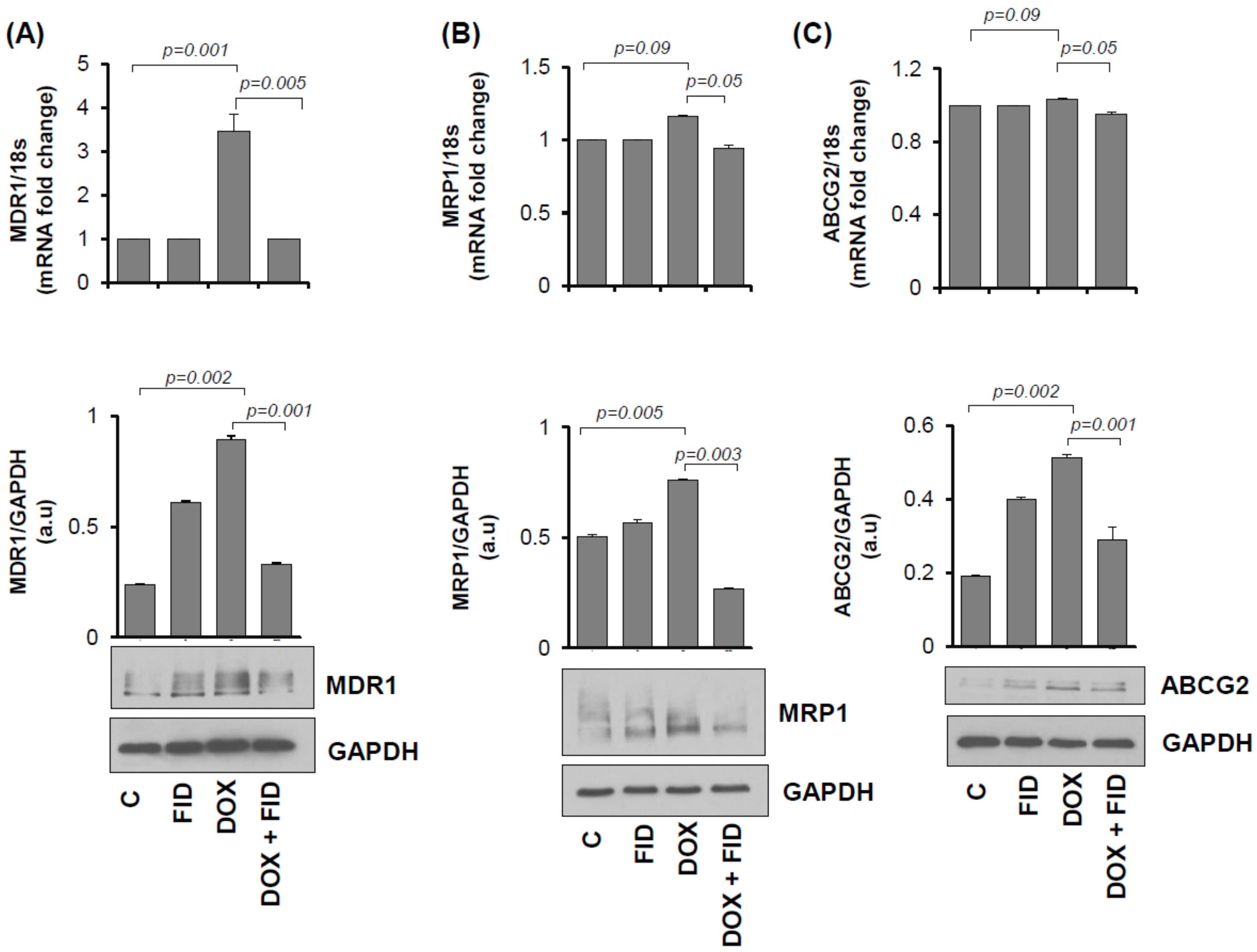

Supplementary Figure.2: Expression of drug transporters in SW480 colon cancer cells: Gene and protein expression levels of drug transporters (A) MDR1 (B) MRP1 and (C) ABCG2 in SW480 CRC cells after 24h of treatment with DOX (1µM) without or with fidarestat (30µM). The representative cropped blots are shown and the full-length blots are presented in Supplementary Fig.8. Individual *p* values are mentioned in the figures. Values are Mean±SD (n=3)

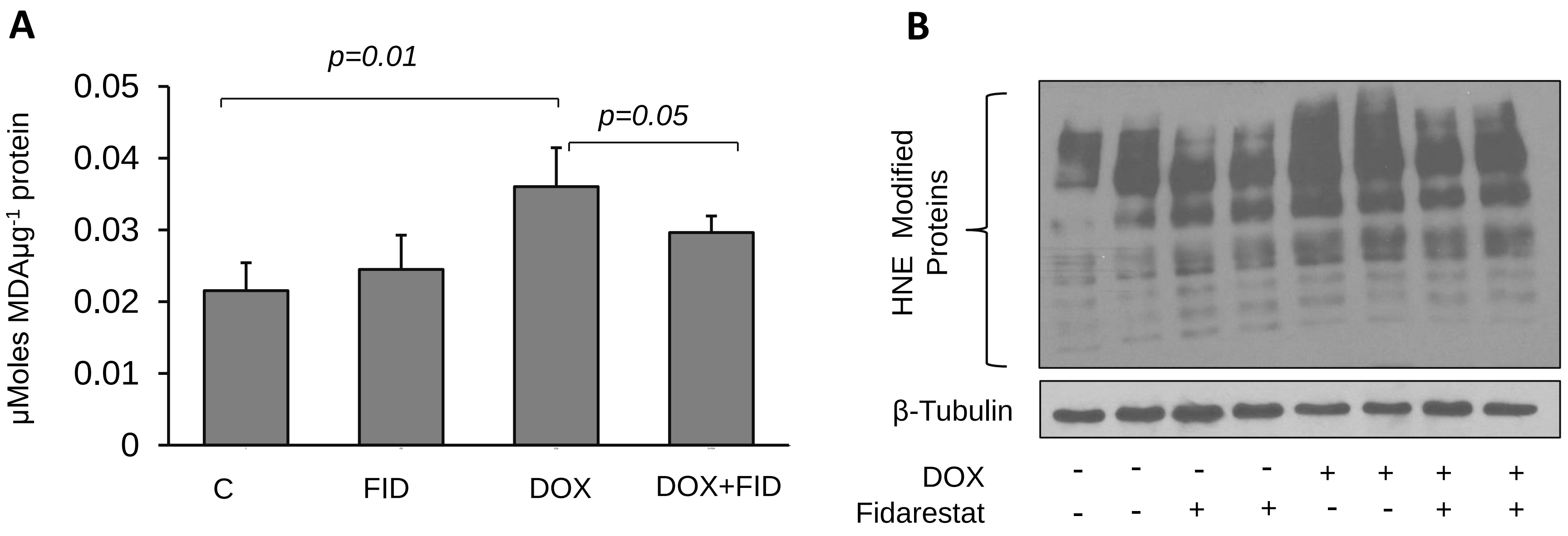

Supplementary Figure 3 : A) MDA levels B) HNE-Modified proteins in heart tissue of mice: Nude mice were treated with DOX (4mg kg^{-1}) alone or in combination with fidarestat (25mg kg^{-1}) for 21 days. A) Levels of MDA in heart tissue homogenates were determined using MDA assay kit (Oxis Research) and B) protein-HNE conjugated proteins by Western blot analysis. A representative blot is shown (n=6). A cropped blot was shown and a full lenghth images are shown in the Supplementary Fig. 9. Individual p values are indicated in the figures and Values are mean± SD (n=6).

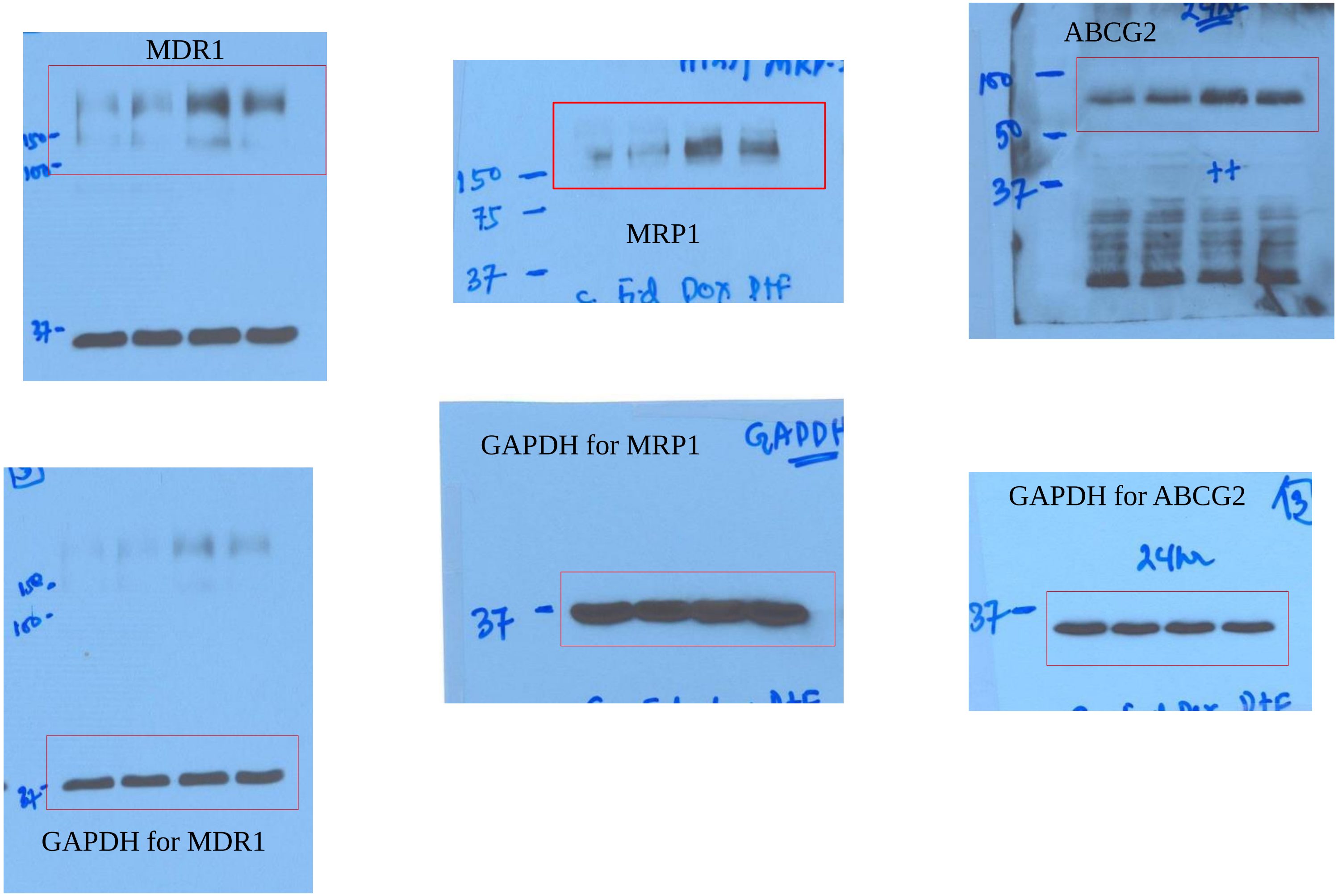

Supplementary Figure 4: Full length blots cropped for representative figures shown in Fig. 3.

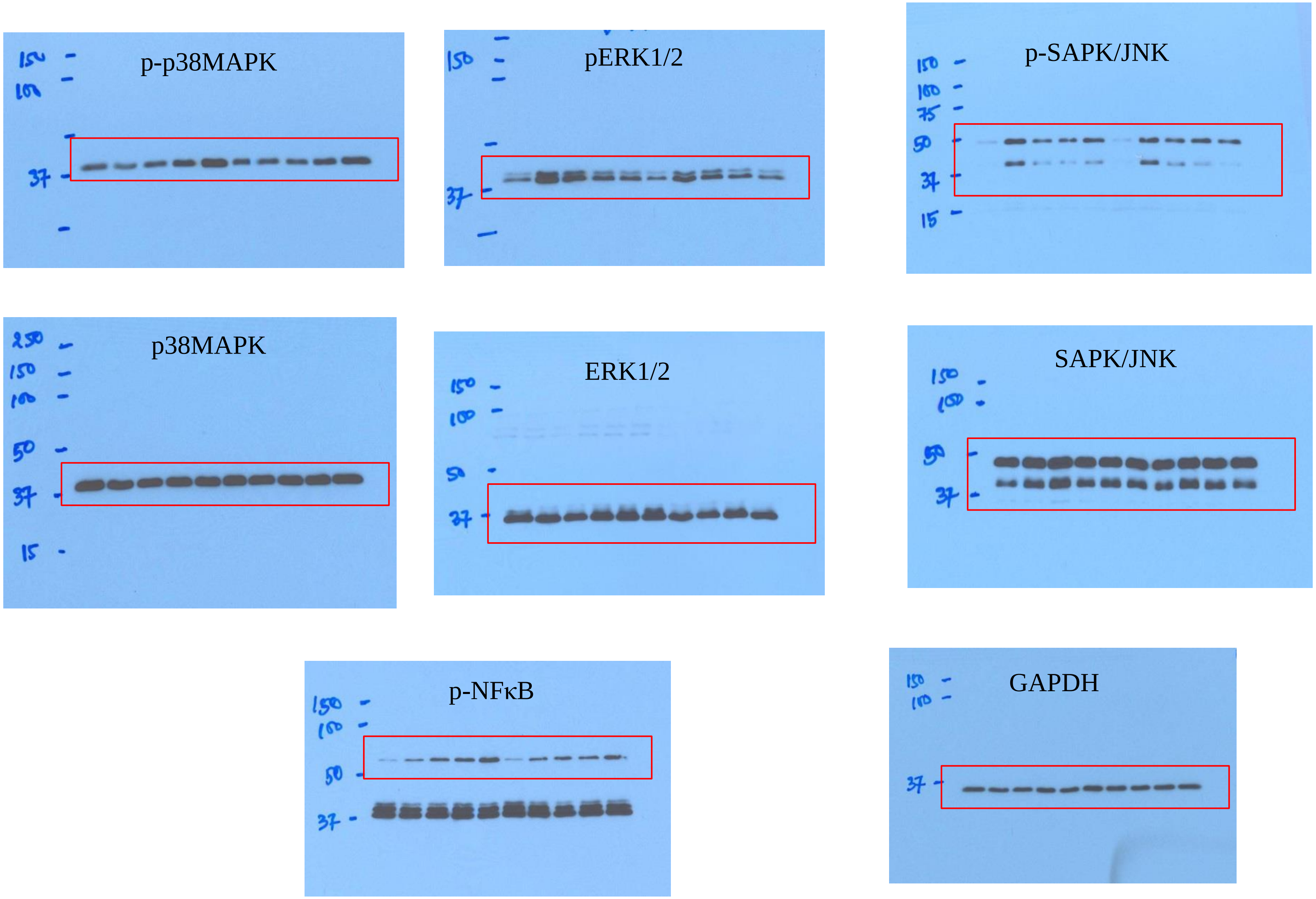

Supplementary Figure 5: Full length blots cropped for representative figures shown in Fig. 4.

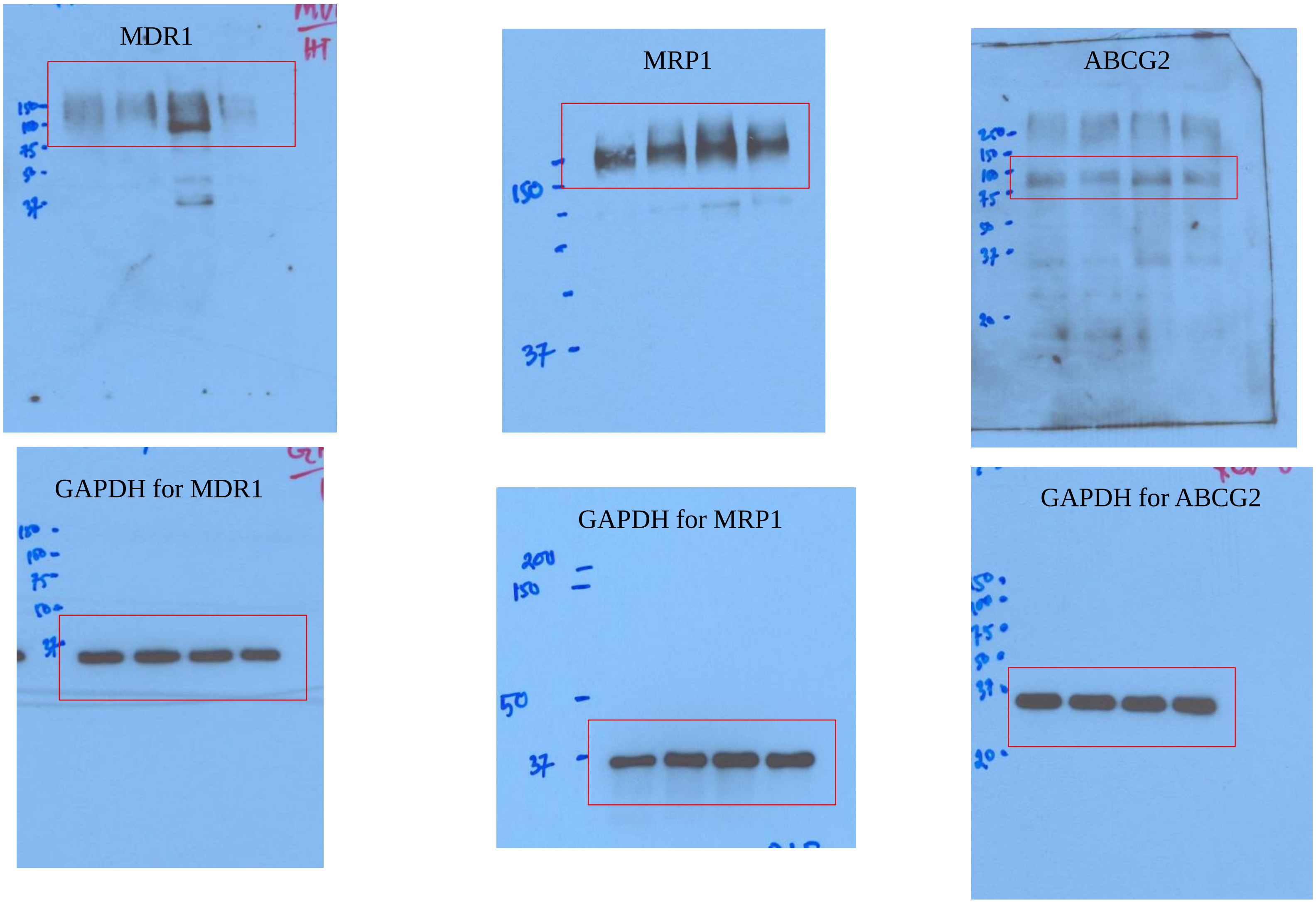

Supplementary Figure 6: Full length blots cropped for representative figures shown in Fig. 5.

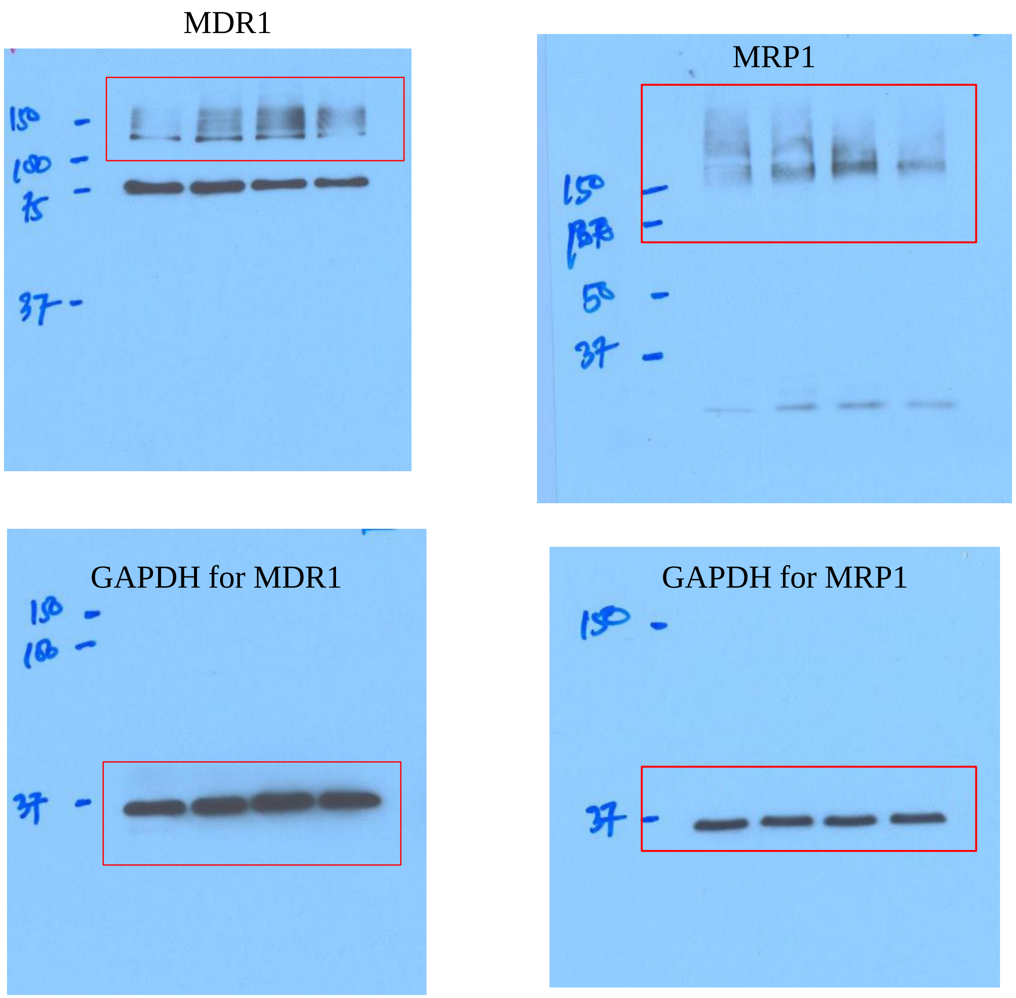

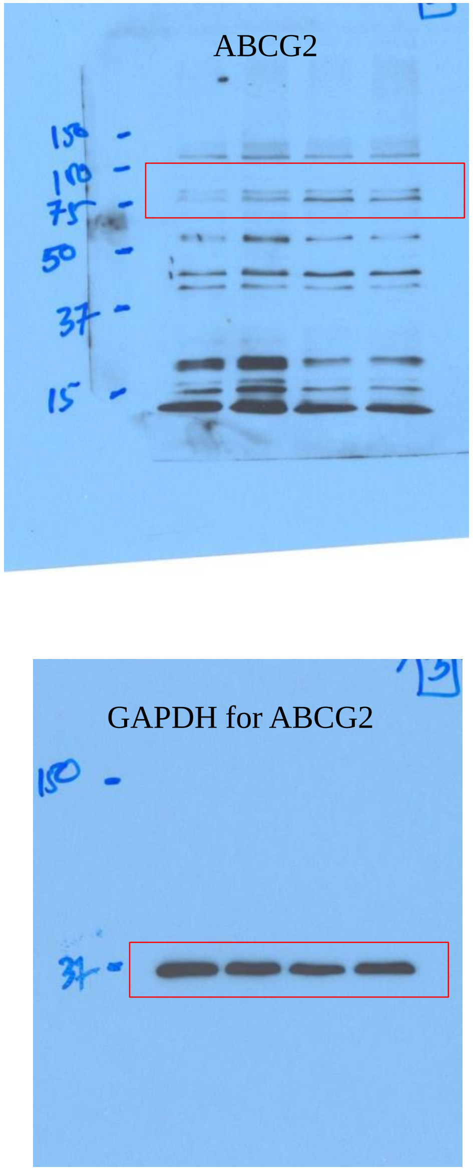

Supplementary Figure 7: Full length blots cropped for representative figures shown in supplementary Fig. 2.

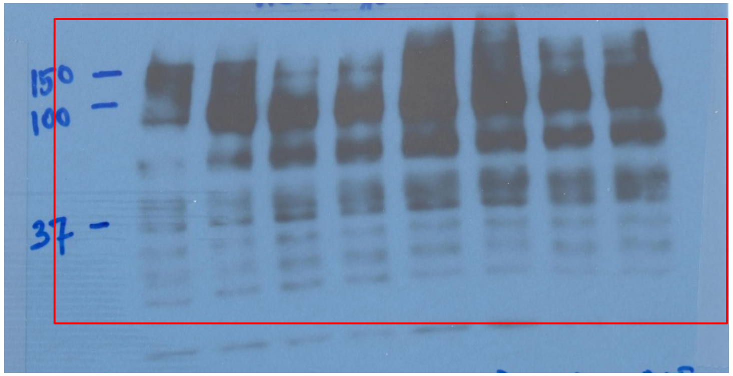

HNE Modified Proteins

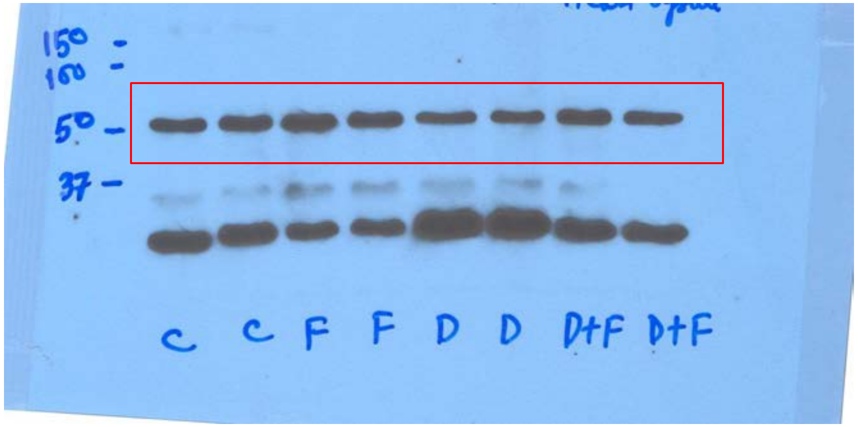

β-tubulin

Supplementary Figure 8: Full length blots cropped for representative figures shown in supplementary Fig.3B.

Supplementary Table 1: Inflammatory cytokines in serum of nude mice treated with DOX alone or in combination with fidarestat

	C	FID	DOX	DOX+FID
IL-1α	159.1±39.9	244.9±80.4	317.5±56.04**	145.7±10.01##
IL-1β	1.7±0.9	1.7±1.1	2.4±0.2*	1.6±1.3#
IL-6	2.5±1.3	ND	5.3±2.3**	6.2±3.2
IL-9	84.6±31.2	86.4±45.09	111.2±58.5	161.1±46.2@
IL-10	2.08±0.2	2.5±1.7	2.5±0.1	3.2±1.7
IL-15	35.2±23.4	17.8±6.5	82.1±10.8*	27.4±6.2##
IP-10	456.4±151.3	443.1±235.4	745.05±167.3*	232.6±32.2##
KC	32.3±21.05	39.7±3.9	33.4±6.8	31.4±8.2
TNFα	1.07±0.3	1.04±0.2	1.7±0.4*	1.5±0.5
G-CSF	275.6±69.2	145.6±17.3	433.8±148.3*	344.5±86.4##

*p< 0.01 vs Control; **p<0.001 vs Control; #p< 0.01 vs DOX; ##p<0.001 vs DOX; @ p<0.01 vs Control; ND= not detectable

Supplementary Table.1: Inflammatory cytokines in serum of nude mice treated with DOX alone or in combination with Fidarestat: Inflammatory cytokines analyzed in serum of nude mice after 21 days of treatment with DOX (4mg kg^{-1} wk^{-1} i.p) alone or with fidarestat in drinking water (25mg kg^{-1}). Blood was collected from individual mouse and serum samples were analyzed by using a mouse inflammatory cytokine/chemokine magnetic bead panel (Milliplex MAP Kit) using a Millipore Milliplex analyzer system. Samples were analyzed in triplicates. Values are Mean±SD (n=6)

Supplementary Table 2: Inflammatory cytokines in heart tissue lysate of nude mice

	C	FID	DOX	DOX+FID
IFNγ	ND	ND	1.02±0.7*	ND
IL-1α	14.4±5.1	16.1±1.2	20.2±5.1**	12.7±1.9#
IL-1β	2.3±0.5	2.2±0.2	3.2±0.1*	1.6±0.4##
IL-2	1.5±0.6	1.9±0.1	2.7±0.6*	1.4±0.3##
IL-6	ND	ND	1.2±0.1**	ND
IL-9	101.4±20.7	111.1±6.7	152.5±25.6*	132.7±21.003
IL-10	1.09±0.1	ND	1.7±0.9*	ND
IL-12 (p40)	5.2±3.1	4.5±0.7	9.5±1.4**	4.1±2.9##
IL-13	ND	5.1±0.5	16.3±5.1*	ND
IP-10	1.1±0.5	0.7±0.1	1.3±0.1*	0.6±0.3##
KC	4.5±0.5	4.06±0.3	7.04±1.1*	4.1±1.1##
LIF	29.4±0.1	40.9±11.4*	65.07±40.6**	8.4±0.1##
LIX	17.1±0.1	48.90±20.1*	27.5±10.7*	ND
MCP-1	ND	ND	2.4±0.1*	ND
M-CSF	9.7±5.6	8.6±4.5	6.3±3.2	ND
MIG	2353.1±1325.4	3564.07±2585.5*	1431.1±531.5*	618.4±317.1##
MIP-1α	3.4±0.7	3.7±0.3	4.6±0.2*	2.7±0.7#
VEGF	595.7±76.2	781.3±344.3*	837.3±186.4*	361.4±107.2#

*p< 0.01 vs Control; **p<0.001 vs Control; #p< 0.01 vs DOX; ##P<0.001 vs DOX; ND= not detectable

Supplementary Table.2: Inflammatory cytokines in heart tissue lysate of nude mice: Expression of inflammatory cytokines in heart tissue lysate of nude mice after 21days of treatment with DOX (4mg kg^{-1} wk^{-1} i.p) alone or in combination with fidarestat in drinking water (25mg kg^{-1}). Values are Mean±SD. Heart tissue were homogenized in MILLIPLEX MAP Lysis Buffer and analyzed by using a mouse inflammatory cytokine/chemokine magnetic bead panel (Milliplex MAP Kit) using a Millipore Milliplex analyzer system. Samples were analyzed in triplicates. Values are Mean±SD (n=6 in each group).
